# Calcium Phosphate Coating Prepared by Microarc Oxidation Affects *hTERT* Expression, Molecular Presentation, and Cytokine Secretion in Tumor-Derived Jurkat T Cells

**DOI:** 10.3390/ma13194307

**Published:** 2020-09-27

**Authors:** Larisa S. Litvinova, Olga G. Khaziakhmatova, Valeria V. Shupletsova, Kristina A. Yurova, Vladimir V. Malashchenko, Egor O. Shunkin, Pavel A. Ivanov, Ekaterina G. Komarova, Valentina V. Chebodaeva, Ekaterina D. Porokhova, Elena A. Gereng, Igor A. Khlusov

**Affiliations:** 1 Center for Immunology and Cell Biotechnology, Immanuel Kant Baltic Federal University, 236029 Kaliningrad, Russia; hazik36@mail.ru (O.G.K.); vshupletsova@mail.ru (V.V.S.); kristina_kofanova@mail.ru (K.A.Y.); vvslon@rambler.ru (V.V.M.); egor.shunkin@gmail.com (E.O.S.); ivanov39pavel@gmail.com (P.A.I.); 2 Laboratory of Physics of Nanostructured Biocomposites, Institute of Strength Physics and Materials Science SB RAS, 634055 Tomsk, Russia; katerina@ispms.ru (E.G.K.); vtina5@mail.ru (V.V.C.); 3 Department of Morphology and General Pathology, Siberian State Medical University, 634050 Tomsk, Russia; porohova_e@mail.ru (E.D.P.); e-gereng@mail.ru (E.A.G.); 4 Research School of Chemistry and Applied Biomedical Sciences, National Research Tomsk Polytechnic University, 634050 Tomsk, Russia

**Keywords:** titanium substrate, CaP roughness, surface electrostatic and electrokinetic potentials, cell behavior in vitro, correlations

## Abstract

Calcium phosphate (CaP) materials are among the best bone graft substitutes, but their use in the repair of damaged bone in tumor patients is still unclear. The human Jurkat T lymphoblast leukemia-derived cell line (Jurkat T cells) was exposed in vitro to a titanium (Ti) substrate (10 × 10 × 1 mm^3^) with a bilateral rough (average roughness index (*R_a_*) = 2–5 μm) CaP coating applied via the microarc oxidation (MAO) technique, and the morphofunctional response of the cells was studied. Scanning electron microscopy (SEM), X-ray diffraction (XRD), and energy dispersive X-ray spectroscope (EDX) analyses showed voltage-dependent (150–300 V) growth of structural (*R_a_* index, mass, and thickness) and morphological surface and volume elements, a low Ca/P_aT_ ratio (0.3–0.6), and the appearance of crystalline phases of CaHPO_4_ (monetite) and β-Ca_2_P_2_O_7_ (calcium pyrophosphate). Cell and molecular reactions in 2-day and 14-day cultures differed strongly and correlated with the *Ra* values. There was significant upregulation of *hTERT* expression (1.7-fold), IL-17 secretion, the presentation of the activation antigens CD25 (by 2.7%) and CD95 (by 5.15%) on CD4^+^ cells, and 1.5–2-fold increased cell apoptosis and necrosis after two days of culture. Hyperactivation-dependent death of CD4^+^ cells triggered by the surface roughness of the CaP coating was proposed. Conversely, a 3.2-fold downregulation in *hTERT* expression increased the percentages of CD4^+^ cells and their CD95^+^ subset (by 15.5% and 22.9%, respectively) and inhibited the secretion of 17 of 27 test cytokines/chemokines without a reduction in Jurkat T cell survival after 14 days of coculture. Thereafter, cell hypoergy and the selection of an *hTERT-*independent viable CD4^+^ subset of tumor cells were proposed. The possible role of negative zeta potentials and Ca^2+^ as effectors of CaP roughness was discussed. The continuous (2–14 days) 1.5–6-fold reductions in the secretion of vascular endothelial growth factor (VEGF) by tumor cells correlated with the *R_a_* values of microarc CaP-coated Ti substrates seems to limit surgical stress-induced metastasis of lymphoid malignancies.

## 1. Introduction

Calcium phosphate (CaP) materials are among the best bone graft substitutes because they promote rapid bone formation and remodeling within their volume and on their surface and may ensure bone healing within a year. Despite these achievements, the mechanisms behind the CaP features (structure, size, and solubility) that induce healthy bone formation are still incompletely understood [1]. Moreover, the human population is aging, and the rates of osteoporosis (up to 30% of hospital beds) [1] and cancer patients are escalating. Clinical success was already achieved with CaP-coated metallic implants in the 1980–90s [2].

Plasma electrolytic or microarc oxidation (MAO) is one the most applicable techniques to prepare metal-oxide and CaP coatings [3,4,5,6] that can be relatively easily improved and innovate traumatological and orthopedic practice. We have previously detected the synthetic microterritories in microarc CaP coating which promote osteogenic differentiation and maturation of mesenchymal stem cells in vitro [7]. However, a question still remains: Are CaP materials a good choice for repairing damaged bone in tumor patients?

The CaP materials are used to treat bone cysts [8] caused by an oncotomy in particular. The key problem is to overcome the minimal residual disease and to kill malignant mesenchymal or leukemic cells in the highly vascularized bone and red marrow (RM) areas [9]. Bone and RM could create a very efficient niche to support the survival and proliferation of the healthy and tumor cells [10], including metastatic cancer cells [11]. Aveic et al. (2019) concluded that CaP porous material as a simplified three-dimensional (3D) replica of bone and RM niche provides a mimetic 3D niche suitable for invasive tumor cells [11].

Therefore, scaffold-based strategy is one of the modern platforms to simulate the physical-chemical, mechanical, and structural traits of the extracellular matrix microenvironment (stiffness, topography, elemental and phase composition, solubility, etc.) that is useful for in vitro engineering and studying of solid and non-solid tumors [12,13]. There were significant attempts to optimize bone and marrow niche models for leukemic cells [14]. Biodegradable and non-biodegradable polymers, such as poly (l-lactic-co-glycolic acid), polyurethane, poly (methyl- methacrylate), poly (d,l-lactide), poly (caprolactone), and polystyrene, have been used in both in vitro [13,15] and in vivo [16] models to investigate acute myeloid leukemia and multiple myeloma, mainly. According to unresolved questions in humanized tumor ECM modeling reviewed in [13], artificial material should be as biomimetic as possible. Certainly, CaP-based approaches must contribute main efforts for bone and RM engineering in pathological conditions. However, several publications were devoted to a CaP-based technique to control a behavior of human malignant hematopoietic cells [11,17].

Hereafter, malignant cell lines, such as MG-63 osteoblast-like cells [18] and human leukemic T lymphoblast-like cell line (Jurkat T cells) [19,20], are often used for orthopedic material testing to develop rough titanium (Ti) and TiO_2_ surfaces. Jurkat T cells were specifically obtained to screen calcium signaling in T cell receptor (TCR)-CD3-CD4 activation [21] and triggered cell-cycle progression in resting T cell populations [22]. Therefore, these cells may be applicable targets for testing the effect of CaP on leukemic cells. Numerous in vitro studies describing mainly short-term (typically no more than 72 h) cultures [19,20,23,24,25,26] provide incomplete information about malignant cell progression and interactions with artificial materials designed for longitudinal contact with the body. Only single articles described a long-term influence of certain CaP materials on some types of cancer cells. For example, the study in [11] explored a behavior (proliferation, survival, and differentiation) of neuroblastoma cells metastasized to bone and RM and contacted in vitro for 10 days with the β-tricalcium phosphate porous scaffolds.

Hence, this investigation aimed to examine the short-term (2 days) and prolonged (14 days) in vitro morphofunctional reactions of Jurkat T cells induced by a rough CaP coating prepared via the MAO method on a Ti substrate.

## 2. Materials and Methods

### 2.1. Sample Preparation and MAO Treatment Procedures

Pure, commercial Ti (Grade 2) billets with a thickness of 1.0 mm (VSMPO-AVISMA Corp., Verkhnaya Salda, Russia) were used as the initial material. The Ti was cut into plates with a size of 10 × 10 mm^2^ and polished with SiC paper with a grade ranging from P180 to P2000. Then, the samples were ultrasonically cleaned (Elmasonic S, Elma Schmidbauer GmbH, Singen, Germany) in distilled water and ethanol for 10 min and dried in air.

Microarc 3.0 installation (ISPMS SB RAS, Tomsk, Russia) was used to carry out the MAO process [27]. The installation system consisted of a pulsed DC power supply, an electrolytic bath with a water-cooling system, two electrodes, and a computer to control the process. To synthesize the MAO coatings, the standard electrolyte contained 27 wt.% H_3_PO_4_, 7 wt.% CaCO_3_, 5 wt.% nanosized stoichiometric hydroxyapatite (HAp; Ca_10_(PO_4_)_6_(OH)_2_) and distilled water as a balance [28]. The obtained electrolyte suspension was an acidic medium with a pH of 1–2. The stoichiometric HAp was produced by the mechanochemical method in the AGO-3 planetary mill at the Institute of Solid State Chemistry and Mechanochemistry of SB RAS (Novosibirsk, Russia) as previously described [29].

The unipolar anodic potentiostatic regime at a fixed pulse frequency of 50 Hz, pulse duration of 100 µs, time of 10 min, and varied voltage from 150 to 300 V with a step of 50 V was used as previously described [30].

### 2.2. MAO Coating Characterization

The morphology, structure, and elemental composition of the coatings were examined by scanning electron microscopy (SEM) on a LEO EVO 50 electron microscope (Carl Zeiss, Oberkochen, Stuttgart, Germany) equipped with an energy dispersive X-ray spectroscope (EDX, INCA, Oxford Instruments, High Wycombe, UK). EDX microanalysis was performed in the microareas of the coating surface on SEM images. The coating thickness was measured by the secant method using SEM images according to ASTM E1382-9 and DD ENV 1071-5 standard protocols.

The phase composition was determined with X-ray diffraction (XRD, XRD 6000, Shimadzu Corp., Kyoto, Japan) in the angular range of 2*θ* = 10–80° with a scanning step of 0.02° and counting time of 3 s per step using Cu Kα radiation. The qualitative phase analysis of the XRD patterns was performed using the ICDD PDF 4+ database.

The mass of the experimental samples before and after MAO treatment was measured using a digital microanalytical balance (GR-202, A&D Company, Tokyo, Japan). The surface roughness was estimated by the average roughness (*R_a_*) using a contact profilometer (Hommel-Etamic T1000 Basic, Jenoptik, Jena, Germany) [31] because a strong linear correlation between *R_a_*, *R_z_*, and *R_max_* for microarc CaP coatings was identified [28]. The traverse length and rate of the measured profile were 6 mm and 0.5 mm/s, respectively. Ten randomly selected traces were recorded for each specimen.

The equipment for the SEM and EDX studies was provided by the “Nanotech” Common Center for Collective Use (ISPMS SB RAS, Tomsk, Russia), and equipment for XRD analysis was provided by the Tomsk Materials Science Center for Collective Use (National Research Tomsk State University, Tomsk, Russia).

The Eguchi method (the method of lifting the electrode) [32] was used to measure the electrical potential (EP) of the CaP surface under ambient conditions and is described in detail in [28].

The electrokinetic (ζ) potential was determined at Far East Federal University (Vladivostok, Russia) using a Z-potentiometer for solid surface analysis (SurPASS 3, Anton Paar GmbH, Graz, Austria) in 0.05 M KCl aqueous solution as described in [33].

Before biological analyses, the CaP-coated Ti samples were sterilized using dry heat with a Binder FD53 (Binder GmbH, Tuttlingen, Germany) at 453 K for 1 h.

### 2.3. Cell Culture

Jurkat T cells were established in 1976 and were isolated from the peripheral blood of a 14-year-old boy with acute lymphoblastic leukemia; these cells were received from the Cell Bank of the Institute of Cytology (Institute of Cytology, Russian Academy of Sciences, Saint Petersburg, Russia) and were cultured at a density of 2 × 10^6^ viable cells per 1.5 mL of nutrient medium consisting of 90% RPMI-1640 (Sigma-Aldrich, St. Louis, MO, USA), 10% inactivated (for 30 min at 56 °C) fetal bovine serum (Sigma-Aldrich, St. Louis, MO, USA), 0.3 mg/mL L-glutamine (Sigma-Aldrich, St. Louis, MO, USA), and 100 U/mL penicillin/streptomycin (Sigma-Aldrich, St. Louis, MO, USA). The initial culture consisted of 96% living, 1% apoptotic, and 3% necrotic cells, as shown via flow cytometry (FC) using propidium iodide (PI, Sigma Aldrich, St. Louis, MO, USA) and Annexin V–fluorescein isothiocyanate (FITC) (Abcam, Cambridge, UK) with a MACS Quant FL7 system (Miltenyi Biotec, Bergisch Gladbach, Germany). Each well of a 12-well flat-bottom plate (Orange Scientific, Braine-l’Alleud, Belgium) was filled with one CaP-coated Ti substrate. Six or seven samples per group prepared at different applied voltages were used in 14-day and 2-day cultures, respectively. A cell suspension without test samples was used as a control. The cell cultures were incubated for 2 or 14 days in a humidified atmosphere of 95% air and 5% CO_2_ at 37 °C. While the cells were cultured for 14 days, the nutrient medium was replaced with fresh medium every 3–4 days.

After culture, the cell suspension was centrifuged at 500 g for 10 min. The cell pellet was collected to measure *hTERT* gene expression, apoptosis, necrosis, and membrane antigen presentation. The cell culture supernatants were used to measure spontaneous and CaP coating-induced secretion. In vitro manipulation was approved by the Local Ethics Committee of Innovation Park, Immanuel Kant Baltic Federal University, Kaliningrad, Russia (Permission No. 2 from 6 March 2017).

### 2.4. hTERT Expression in Cells

To determine the expression of *hTERT* (the telomerase reverse transcriptase gene) in Jurkat T cells, mRNA was extracted using an RNeasy mini kit (Qiagen, Hilden, Germany) according to the manufacturer’s instructions. Total RNA (100 ng) was subsequently reverse-transcribed into cDNA using the QuantiTect reverse transcription kit (Qiagen, Hilden, Germany). *hTERT* transcription was analyzed via SYBR Green-based quantitative polymerase chain reaction (PCR) as described previously [9]. The results are expressed in arbitrary units as the ratio between the relative amount of *hTERT* cDNA and the relative amount of 18S rRNA cDNA. 18S rRNA cDNA did not exhibit significant changes in expression and was used as a housekeeping gene [34].

### 2.5. Measurement of Cell Secretion

Flow fluorimetry (FF) was performed to measure the concentrations (pg/mL) of the following human cytokines and chemokines: interleukin (IL)-1β, IL-1Ra, IL-2, IL-4, IL-5, IL-6, IL-7, IL-8, IL-9, IL-10, IL-12, IL-13, IL-15, IL-17, tumor necrosis factor alpha (TNFα), interferon gamma (IFN-γ), basic fibroblast growth factor (FGFb), platelet-derived growth factor (PDGF-BB), vascular endothelial growth factor (VEGF), granulocyte colony-stimulating factor (G-CSF), granulocyte-macrophage colony-stimulating factor (GM-CSF), eotaxin, interferon gamma-induced protein 10 (IP-10; C-X-C motif chemokine 10 (CXCL10), monocyte chemoattractant protein-1 (MCP-1; chemokine (C-C motif) ligand 2 (CCL2), and macrophage inflammatory protein 1 alpha (MIP-1α; CCL3), MIP-1β (CCL4), regulated upon activation, and normal T cell expressed and secreted (RANTES; CCL5). FF was performed with monoclonal antibodies (mAbs) according to the manufacturer’s instructions for the cytokine assay system (Bio-Plex Pro Human Cytokine 27-Plex Panel, Bio-Rad, Hercules, CA, USA) using an automated processing system (Bio-Plex Protein Assay System, Bio-Rad, Hercules, CA, USA).

### 2.6. Cell Viability and Immunophenotype Detection

The fluorescent staining methods more accurately estimate cell viability than trypan blue because ruptured dead or dying Jurkat cells cause an overestimation of the number of live cells when the viability falls below 80% [35]. Therefore, the in vitro viability of Jurkat T cells was estimated with a MACS Quant flow cytometer (Miltenyi Biotec, Bergisch Gladbach, Germany). After culture, the cells were resuspended in binding buffer, and 5 µL of Annexin V-FITC (Abcam, Cambridge, MA, USA) was added to 195 µL of the cell suspension. The cells were incubated for 10 min, washed and resuspended in binding buffer. An aliquot of 190 µL of the cell suspension was mixed with 10 µL of PI solution (Abcam, Cambridge, MA, USA), and the resulting mixture was analyzed by flow cytometry (FC). The percentages of live and dead (apoptotic or necrotic) cells were measured according to the manufacturer’s protocol.

Alizarin red S (ARS, Sigma-Aldrich, St. Louis, MO, USA) staining to detect calcification by intracellular diffusion into adherent Jurkat T cells was performed as recommended by the manufacturer after 14 days of culture.

The cell immunophenotype was analyzed using specific mAbs (see below) according to the manufacturer’s instructions. The mAbs were labeled with FITC, allophycocyanin (APC), phycoerythrin (PE), violet blue (VioBlue), or phycoerythrin cyanine 7 (PE-Cy7) (Table 1). Jurkat T cells were washed with phosphate-buffered saline (pH = 7.2), and the cell suspension was mixed with a cocktail of mAbs against CD45, CD3, CD4, CD8, and CD25 (Abcam, Cambridge, UK) and CD45RO, CD45RA, CD71, and CD95 (e-Bioscience, San Diego, CA, USA). The algorithm and gating strategy for CD45^+^CD3^+^ subpopulation cytometry were previously described [20].

After a 10 min incubation with the labeled mAbs, the cells were assayed using a MACS Quant flow cytometer (Miltenyi Biotec, Bergisch Gladbach, Germany) according to the manufacturer’s protocol. FC results were analyzed using KALUZA analysis software (Beckman Coulter, Brea, CA, USA).

### 2.7. Statistical Analysis

Statistical analyses were conducted using the STATISTICA 13.3 software package for Windows. The mean (X) and standard deviation (SD) or median (Me) and 25% (Q1) and 75% (Q3) quartiles were calculated. The normality of the data distribution was determined by the Kolmogorov-Smirnov test. Because of the nonnormal data distribution, the nonparametric Mann–Whitney *U* test was performed, and statistically significant differences were considered at *p* < 0.05. The relationships between the analyzed parameters were established via correlation (Spearman) analyses. Significant relationships were indicated by coefficient (r) values with a significance level greater than 95%.

## 3. Results

### 3.1. Morphology, Stucture, and Properties of CaP Coatings

**Figure 1:**
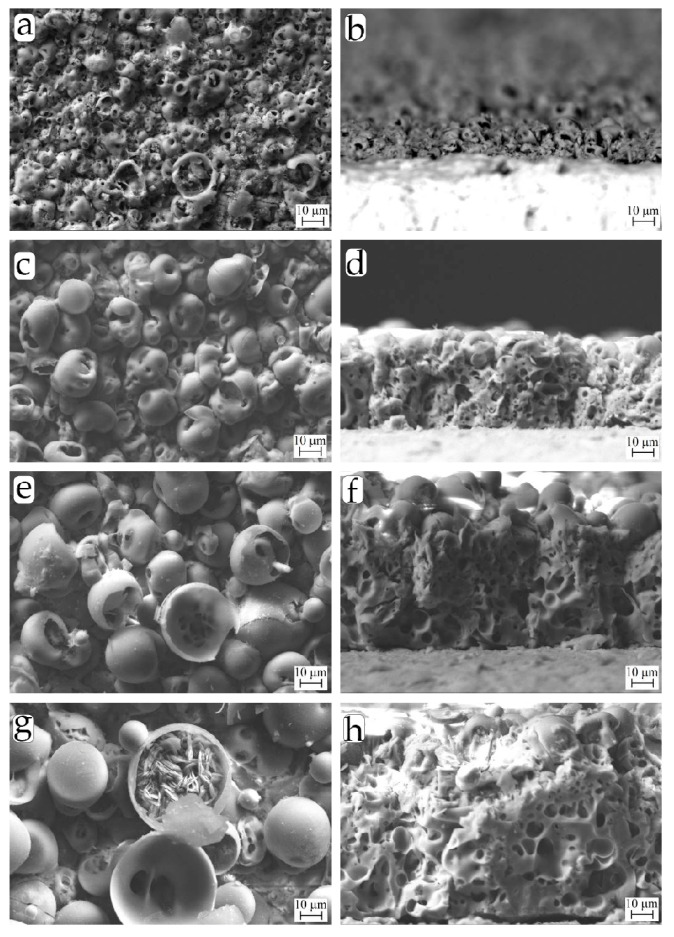
Scanning electron microscopy (SEM) images of the surface (**a**,**c**,**e**,**g**) and cross-section (**b**,**d**,**f**,**h**) of the CaP coatings deposited at 150 V (**a**,**b**), 200 V (**c**,**d**), 250 V (**e**,**f**), and 300 V (**g**,**h**).

SEM images represent the surface and cross-section morphology of the CaP coatings deposited by the MAO method under the different applied voltages (Figure 1). As can be seen, the morphology and thickness of the coatings significantly depend on the value of the applied voltage. At the lowest voltage of 150 V, the initial nucleation of hemispherical-shaped structural elements with internal pores on the coating surface occurs (Figure 1a). The thickness of this coating does not exceed 20 µm (Figure 1b).

With an increase in the applied voltage from 200 V to 250 V, spheroidal structural elements (spherulites) and hemispheres with internal pores were completely formed on the coating surface (Figure 1c,e). In addition to internal pores, external pores between the spherulites were observed. Spherulite nucleation occurs by the formation and collapse of a vapor-gas/plasma bubble during the MAO process [36,37]. The coatings deposited at 200–250 V had thicknesses of 50–80 µm (Figure 1d,f).

An increase in the applied voltage from 250 to 300 V led to an increase in the intensity of microplasma discharges affecting the substrate. As a result, the coating thickness grew from 80 to 100 µm, the sizes of spherulites and pores increased, and the spherulites were partially destroyed (Figure 1g,h). Moreover, numerous plate-shaped crystals (up to 15 µm in length) formed inside the destroyed hemispheres (Figure 1g).

SEM images of the coating cross-sections show that structural elements such as spherulites, hemispheres, and plate-shaped crystals were contained only in the coating surface (Figure 1b,d,f,h). The inner structure of the coatings included multiple branched microsized pores and pore channels (0.5–30 µm in size) inhomogeneously distributed through the coating thickness.

**Figure 2:**
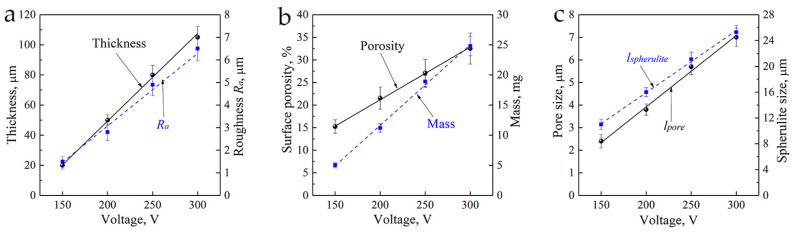
Plots of the coating thickness and roughness, (**a**) surface porosity and mass, and (**b**) size of the spheres and pores (**c**) against the microarc oxidation (MAO)-applied voltage.

An increase in the applied voltage from 150 to 300 V led to a linear increase in the CaP coating characteristics (r > 0.9; *p* < 0.001). Increases in the thickness from 20 to 100 µm, mass from 3 to 25 mg, *R_a_* from 1.5 to 6.5 µm, surface porosity from 15% to 32%, and sizes of the structural elements (the average size of spherulites from 11.0 to 25.3 µm, the average size of pores from 2.4 to 7.0 µm) occurred (Figure 2).

**Figure 3:**
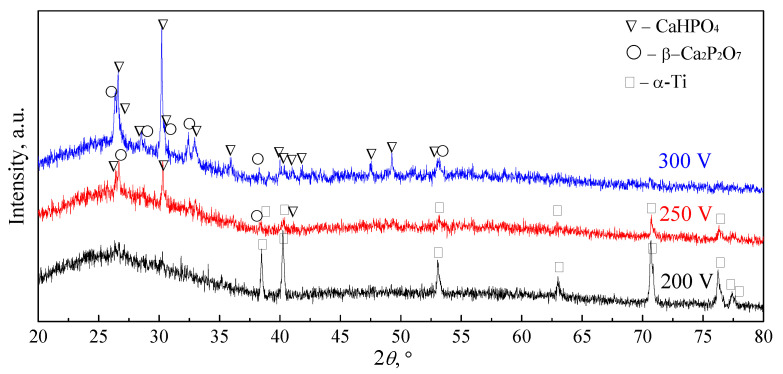
X-ray diffraction (XRD) patterns of the CaP coatings formed at 200 V, 250 V, and 300 V.

XRD studies revealed that the CaP coatings formed at low voltages of 150–250 V were mainly in the X-ray amorphous state as evidenced by the appearance of a diffusive halo at small angles (2θ = 20–38°) and weak reflections from a single Ti phase (ICDD #44-1294) of the substrate in the corresponding XRD patterns (Figure 3). An increase in the MAO voltage led to the coating structure transforming from the X-ray amorphous state into the amorphous-crystalline state. XRD patterns of the coatings formed at high voltages of 250–300 V included diffuse halos, as well as reflections of the crystalline phases of dicalcium phosphate anhydrous (DCPA, monetite, CaHPO_4_) (ICDD #09-0080) and β-calcium pyrophosphate (β-CPP, β-Ca_2_P_2_O_7_) (ICDD #09-0346) (Figure 3). The intensity of the reflections of these CaP phases increased with increasing voltage. These XRD data were consistent with the SEM results, indicating the incorporation of plate-shaped crystals in the coatings formed at high voltages of 250–300 V (Figure 1e,g).

**Figure 4:**
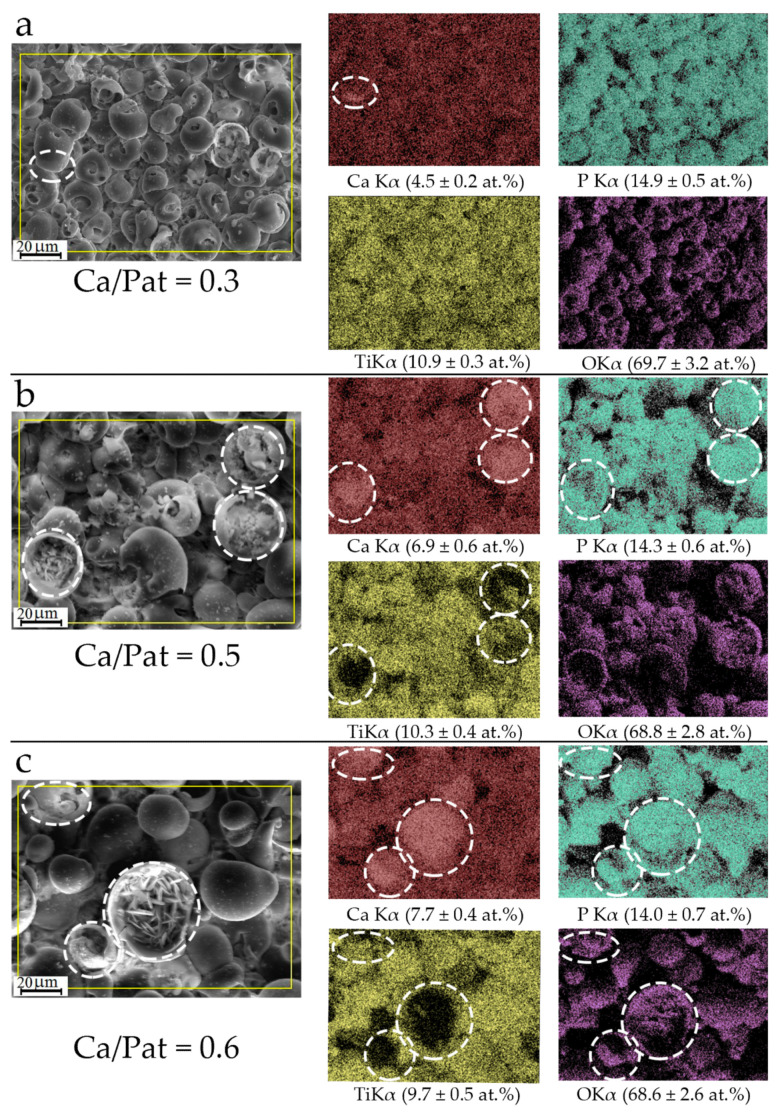
SEM images and EDX gray-level maps of the Ca, P, Ti, and O distributions over the surface of the CaP coatings deposited at 200 V (**a**), 250 V (**b**), and 300 V (**c**).

Figure 4 shows the SEM images and EDX maps of the distribution of the chemical elements (calcium, phosphorus, titanium, and oxygen) over the coating surface. EDX gray-level maps showed that all elements (Ca, P, Ti, and O) were distributed fairly uniformly over the surface of the coating formed at low voltages of 150–200 V (Figure 4a). With increasing applied MAO voltage, the Ca content in the coatings increased from 4.5 to 7.7 at.%, and in contrast, the P, Ti, and O amounts slightly decreased. Increasing the Ca concentration in the coatings led to an increase in the Ca/P atomic ratio from 0.3 to 0.6. The corresponding maps showed that the increased Ca was mainly concentrated inside the destroyed hemispheres with plate-shaped crystals (these areas are marked by white circles in Figure 4b,c). However, the EDX maps show that the oxygen and phosphorus distribution reproduced the rough topography of the coating (*R_a_* = 5.0–6.5 μm).

While CaP coatings obtained by the MAO method are dielectrics, their electrical surface charge is an important feature [28]. The MAO coatings, including the CaP amorphous phase and crystalline DCPA and β-CPP phases, can have both a positive charge in the form of Ca^2+^ ions and a negative charge in the form of phosphate (PO_4_^3−^) and hydroxyl (OH^−^) groups.

**Figure 5:**
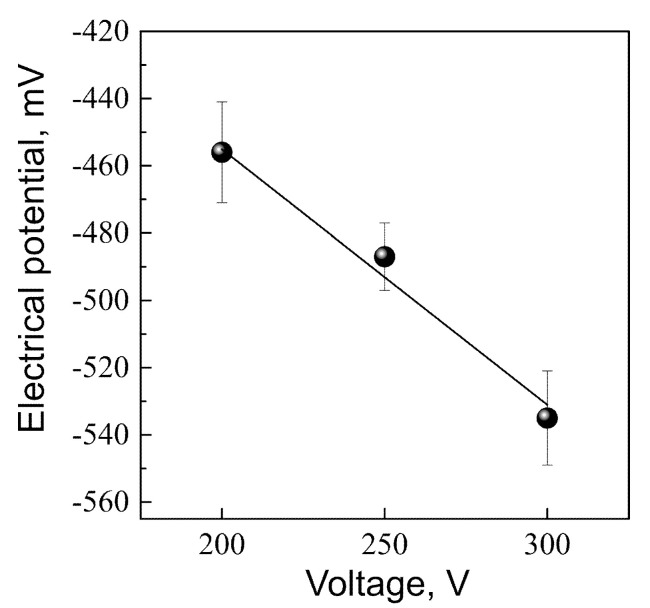
Graph of the electrical potential on the CaP coating surface against the applied MAO voltage.

Studies of the integral EP using the Eguchi method showed that all coatings deposited at the applied voltages had a negative electrical charge on their surface. The amplitude of the EP on the coating surface increased with increasing MAO voltage (Figure 5). This effect can be due to the increase in the surface roughness *R_a_* from 2.5 to 6.5 μm and, consequently, the increase in the free surface area. As a result, the number of negatively charged hydroxyl and phosphate groups increased on the surface of the coatings. These results are consistent with the EDX microanalysis data, which revealed a low Ca/P atomic ratio (0.3–0.6 at.%) in the coatings due to the low calcium content (Figure 4).

Thus, we established the range of the applied MAO voltage of 150–300 V provided to form the MAO coatings with a combination of properties required usually for biological applications. The applied voltage of more than 250 V leads to dramatic decrease in the adhesion strength of thick CaP coating to Ti substrate [5] that limits its employment in orthopedic practice. Therefore, the range of the applied MAO voltage of 150–250 V was used to deposit the coatings as follows: Ca/P atomic ratio of 0.3–0.5, thickness of 24–80 µm, mass of 5–25 mg, and roughness index *R_a_* of 2.0–5.0 µm.

### 3.2. Biological Properties of CaP Coating

In this study, a strong correlation (*r* = 0.94; *n* = 6; *p* < 0.005) was identified between the *R_a_*, thickness, and mass values of the microarc CaP coating (Figure 2), which corresponded to our previous data [28]. Therefore, *R_a_* = 2.0–5.0 µm was mainly used to characterize the relations between the CaP-coated substrates and tumor cell behavior in vitro. Such range of surface roughness is optimal to trigger osteoblastic differentiation and maturation of health stromal cells in vitro [28].

Tumor-derived Jurkat T cells in contact with the rough CaP coating for 2 days showed a 1.7-fold upregulation in *hTERT* expression (Table 2).

No correlations between *R_a_* and gene expression were observed. The majority (98%) of viable Jurkat cells expressed CD3, as expected. CD3^+^ cells in the 2-day control culture expressed the CD4^+^ (95%) and CD71^+^ (75%) profile of CD45RA^+^ naïve cells (not activated by the antigen, 92%), with subsets of CD4^+^ T helpers expressing markers of early (17% of the CD25 subset) and late (83% of CD95^+^ cells) activation and apoptosis (Table 3). Spontaneous secretory activity in 2-day Jurkat T cell cultures was low (concentrations of 27 molecules less than 100 pg/mL, Table 4), as previously described [38].

Short-term exposure of the cells to the CaP relief surface increased the expression of CD45RO (from 1.8% to 7%, *p* < 0.04), which was specific to stimulated T cells (Table 3). This expression was accompanied by the elevated presentation of CD8, which is associated with T cell differentiation and maturation (from 1.44% to 3.6%, *p* < 0.003), and CD25 (by 2.7%) and CD95 (by 5.15%), which are activation antigens on CD4^+^ cells. In addition, significant production of IL-17, GM-CSF, and some chemokines (MCP-1, MIP-1α, and MIP-1β) induced by 2-day contact with the CaP relief surface occurred. At the same time, there was a 1.5-fold decrease in VEGF levels, while the CaP coating influenced Jurkat T cells (Table 4). In turn, the viability of Jurkat T cells diminished significantly, as indicated by 2- and 1.5-fold increases in cell apoptosis and necrosis, respectively, from short-term contact with the CaP-coated Ti samples (Table 3).

It is highly likely that the expression of genes and CD molecules and cell viability induced by short-term contact with CaP-coated samples suggest well-known [39] hyperactivation-dependent death of CD4^+^ cells. We believe that the surface roughness of the CaP coating may be a trigger of the described changes in Jurkat T cell cultures because of the strong associations between *R_a_* indices with increased proportions of necrotic cells (*r* = 0.95, *p* < 0.001, *n* = 10) and the CD4CD95^+^ subset (*r* = 0.86, *p* < 0.001, *n* = 10) and the increased IL-17 (*r* = 0.76, *p* < 0.02, *n* = 9) and MCP-1 (*r* = 0.78, *p* < 0.02, *n* = 9) concentrations. Moreover, a close negative relationship (*r* = −0.78, *p* < 0.02, *n* = 9) between *R_a_* and decreased VEGF secretion was determined.

The rough CaP-coated Ti samples caused a 3.2-fold downregulation of *hTERT* expression in Jurkat T cells after 14 days of culture (Table 2). Overall, there was a direct correlation between *R_a_* values and decreased gene expression (*r* = 0.88, *p* < 0.03, *n* = 6). Long-term contact with the CaP irritant showed increased numbers of CD4^+^ cells and their CD95^+^ subset (by 15.5% and 22.9%, respectively) and diminished proportions of the CD8^+^ and CD71^+^ subpopulations of CD45CD3^+^ cells compared with those of the control 14-day culture (Table 3). The secretion of 17 of the 27 test biomolecules was significantly inhibited (Table 4) without a reduction in Jurkat T cell survival (Table 3). First, the 500-fold and 6-fold downregulated secretion of middle/high levels (more than 0.1 ng/mL) [38] of IL-6 and VEGF, respectively, was caused by the CaP coating. Decreased concentrations of 16 of the 17 cytokines, chemokines, and growth factors correlated with increases in the *R_a_* index in the range of *r* = 0.76–0.88 (*p* < 0.03, *n* = 11).

**Figure 6:**
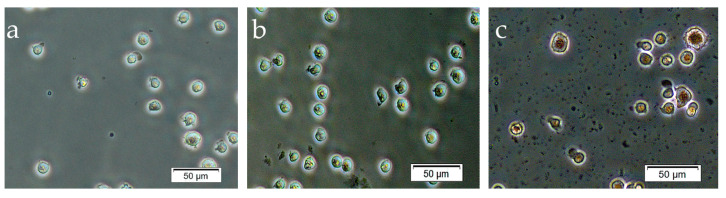
Adherent Jurkat T cells in 14 days of culture either before (**a**) or after alizarin red S (**b**,**c**) staining. (**a**) Unstained cells; (**b**) weakly stained nuclei of cells in the control culture without microarc CaP-coated titanium samples; (**c**) clearly stained nuclei of cells in contact with microarc CaP-coated titanium samples.

In this context, 14-day contact with the CaP-coated Ti samples led to unexpected but clear evidence of enhanced ARS staining of nuclei in adherent Jurkat T cells versus unstained tumor cells in control cultures (Figure 6).

Thus, secretory and gene expression hypoergy of Jurkat T cells was observed after prolonged contact with rough CaP-coated Ti samples, and the close correlation of these effects with the CaP surface roughness index *R_a_* was noted.

## 4. Discussion

Overall, SEM, XRD, and EDX analyses (Figure 1, Figure 2, Figure 3 and Figure 4) showed typical physicochemical properties and some features of the CaP coatings deposited on Ti substrate at voltage ranging from 150–300 V. The features included voltage-dependent growth of structural (*R_a_* index, mass, and thickness) and morphological (spherulites, valleys, and pores) surface and volume elements, calcium content, and the appearance of crystalline phases of CaHPO_4_ (monetite) and β-Ca_2_P_2_O_7_ (calcium pyrophosphate). These findings correspond to our previous results [27] and emphasize the technological reproducibility of the MAO method to produce CaP coatings on Ti substrates.

The bioactive microarc CaP coatings have both direct (surface properties) and indirect effects on cells and tissues via the products of their destruction and biodegradation. The EDX analysis showed low Ca/P ratios (0.3–0.6) in CaP coatings (Figure 4). The release of calcium and phosphate ions from the microarc coatings to a solvent medium was episodic for five weeks of in vitro dissolution [27].

Calcium concentrations in a solvent were insignificant (approximately 0.25 mM per week) [27,40] compared with the high (>2 mM) Ca^2+^ levels measured in other investigations [41,42] due to ion redeposition (precipitation) on the surface of the microarc CaP-coated Ti samples [27].

An increase in surface roughness is accompanied by an increase in the surface area for dissolution. Enhanced extracellular Ca^2+^ levels transiently increase intracellular Ca^2+^ concentrations [43]. Calcium ions are messengers associated with multiple pathways that play an important role in lymphocyte and tumor cells behavior and death [44,45]. According to the results of the present study, the surface roughness of the microarc CaP coating controlled Jurkat T cell cultures. However, due to prolonged and poor dissolution of the surface [40], Ca^2+^ may be only one of the effectors of influence of the microarc CaP coating in vitro. Microparticles (Figure 4), as well as nanocrystallites (~50 nm) of monetite (CaHPO_4_) and calcium pyrophosphate (β-Ca_2_P_2_O_7_), in microarc CaP coatings are capable of extracting [27] and mediating effects on cells. In particular, CaP nanoparticles are internalized and biodegraded by tumor cells [46]. Crystalline calcium pyrophosphate stimulates cell secretion of proinflammatory molecules [47], and pyrophosphate derivatives (bisphosphonates) inhibit tumor cell growth [48].

Surface topography also affects cells [49]. The influence of surface roughness/topography on cells [50] is studied more intensively than the effect of the electrostatic factor of rough dielectric surfaces [28]. However, the balance between the physical and chemical events that affect cell behavior is still unclear.

The morphologies of slightly soluble microarc CaP coatings of different roughness were similar (Figure 1). Hence, the negative EP magnitude was directly dependent on the MAO voltage (Figure 5) and *R_a_* value of the microarc CaP surface [28]. Therefore, its biological significance was also suggested to regulate leukemic Jurkat T cells.

The large magnitude (400–500 mV) of surface EP measured in air (Figure 5) is reduced in electrolytes [33] by the electrokinetic interaction at the artificial surface and solution interface and the formation of a double electric layer [51]. The microarc CaP coating in KCl aqueous solution possessed a negative charge with a zeta (ζ) potential of 63 ± 1 mV, which was 3-fold below its EP measured in air (−202 ± 14 mV). No changes in ζ-potential at pH values in the range of 5.5–8 units at a temperature of 293 K were detected.

The ζ-potential influences negatively charged Jurkat T cell attachment [19], and it is connected with transmembrane potential (TMP) [52]. Tumor cells possess depolarized TMP with a negative charge in the range of 5–50 mV compared with that of quiescent healthy cells that have more negative charges. The TMP is a key biophysical signal in nonexcitable cells that modulates cellular activities, including proliferation, differentiation, viability, and motility [53]. The external electric field impacts the TMP [54], and the reduced resting potential of cells facilitates their sensitivity to irritants. The ζ-potential of the microarc CaP surface is comparable to the TMP of tumor cells and could promote their hyperpolarization or depolarization of the membrane, resulting in an intracellular [Ca^2+^] increase via different types of channels [55].

There were no significant correlations between the adhesion force of negatively charged Jurkat T cells and the roughness of the TiO_2_ negative surface [19]. Regarding calcium release from microarc CaP-coated Ti samples, Ca^2+^ may enhance and prolong Jurkat T cell interactions with negative CaP surfaces (Figure 5) by diminishing electrostatic repulsion. Thereafter, TMP fluctuations during lymphocyte activation also depend on the gradient between the extracellular [Ca^2+^] (~1 mM) and the intracellular [Ca^2+^] (~0.1 μM) [45]. In turn, the increasing extracellular Ca concentration (1 mM CaCl_2_) in the presence of ionomycin induces time-dependent transient changes in the intracellular Ca level and stimulates IL-2 secretion in human Jurkat T cells [44]. The ζ-potential of microarc CaP coatings may promote Ca^2+^ influx by changing the resting potential of cells.

Thus, the roughness of the microarc CaP coating on Ti samples has at least two mediators, namely, the negative surface charge and Ca flux, that affect cells. Therefore, the current study showed no obvious effect of rough CaP-coated Ti samples on Jurkat T cell behavior, unlike the in vitro death of Jurkat T cells that were in short-term contact with a microarc rough TiO_2_ coating generating a negative surface charge. The TiO_2_ effect was accompanied by the downregulation of *hTERT* expression, CD presentation, and cytokine secretion by tumor cells [20].

In contrast, the short-term influence of the microarc rough CaP coating led to clear signs of hyperactivation-dependent death [39] in CD4^+^ leukemic cells expressing CD25 and CD95, which are markers of activation and apoptosis (Table 2, Table 3 and Table 4) possibly triggered by ex vivo irritants. Expression of the telomerase gene (*hTERT*) is crucial for cell survival, and its expression is actively increased in proliferating Jurkat T cells [56] to promote cytokine secretion [57]. Indeed, a 1.7-fold upregulation in *hTERT* expression was observed in Jurkat T cells that were in contact with the CaP coating (Table 2) and was accompanied by an increased number of CD45R0^+^ activated cells (Table 3) and enhanced chemokine secretion (Table 4).

To induce cell death, a sustained influx of extracellular Ca^2+^ and the presence of the death receptor Fas on the target cell have been shown to be required for FasL/Fas-mediated (CD95) cell injury [44]. Overall, increased [Ca^2+^] in the cell nucleus is needed to initiate DNA fragmentation followed by cytolysis [58].

There have been a few reports about ARS staining of intracellular calcium ions, such as in [59]. However, ARS is widely used to analyze extracellular calcium salt deposition, and the difficulties of its uptake into cells are known [60]. Therefore, the results in Figure 6 suggest enhanced membrane permeability caused by Jurkat T cell injury and associated cell death. Indeed, significant downregulation of *hTERT* expression, decreased cytokine and chemokine secretion, and enhanced expression of CD95, a marker of late activation/apoptosis, were detected on CD4^+^ Jurkat T cells after prolonged 14-day contact with microarc CaP coatings (Table 2, Table 3 and Table 4). Chemokines play a crucial role in the metastasis of malignant tumor cells [61] and Jurkat T cells [62]. In addition, VEGF secretion, which was connected with Ca^2+^ influx and promoted angiogenesis-associated tumor cell progression [63], was depleted by 1.5–6 times during the experimental period. At the same time, the CD4^+^ subset expanded from 62% to 78% of the cell population, and cell viability did not fall (Table 3).

Perhaps short-term 2-day culture with CaP induced hyperactivation-dependent death of CD4^+^ Jurkat T cells, followed by a 14-day selection of the *hTERT-*independent viable subsets (Table 2 and Table 3). Reduced expression of the CD71 (Table 3) transferrin receptor stimulated Jurkat T cell proliferation and activation [64] was also noted. Moreover, the cytokine/chemokine network was decreased and included impaired mitotic/costimulatory/antiapoptotic signaling associated with IL-1, IL-4, IL-6, and IL-8 [65,66,67,68,69] (Table 4). Therefore, the molecular mechanisms underlying the 14-day survival of a portion of CD4^+^ Jurkat T cells in the presence of rough and soluble CaP coating have not yet been clarified. Reduced biomolecule production is capable of limiting cytokine/chemokine-induced hyperactivation/death of Jurkat T cells and promoting their survival. The costimulatory cell surface antigen CD28 on Jurkat T cells [70] could be a molecular messenger of the described effect. From a cellular point of view, bone is thought to be a substrate for leukemic cell niches [71,72], and CaP bone minerals may affect the quiescent state of malignant cells.

Regardless, gene (*hTERT*) and secretory (17 of 27 tested biomolecules) hypoergy in tumor immune-competent T cells caused in vitro by long-term contact with microarc rough CaP-coated Ti substrates may be useful for affecting their high progression and damage to bone regeneration after osteosynthesis. We believe that continuously reduced VEGF secretion (Table 4) correlated with the *R_a_* surface index is able to limit surgical stress-induced metastasis of hematopoietic malignancies. These findings need to be verified in inbred animals with spontaneous leukemia, e.g., the AKR line of mice.

**Figure 7:**
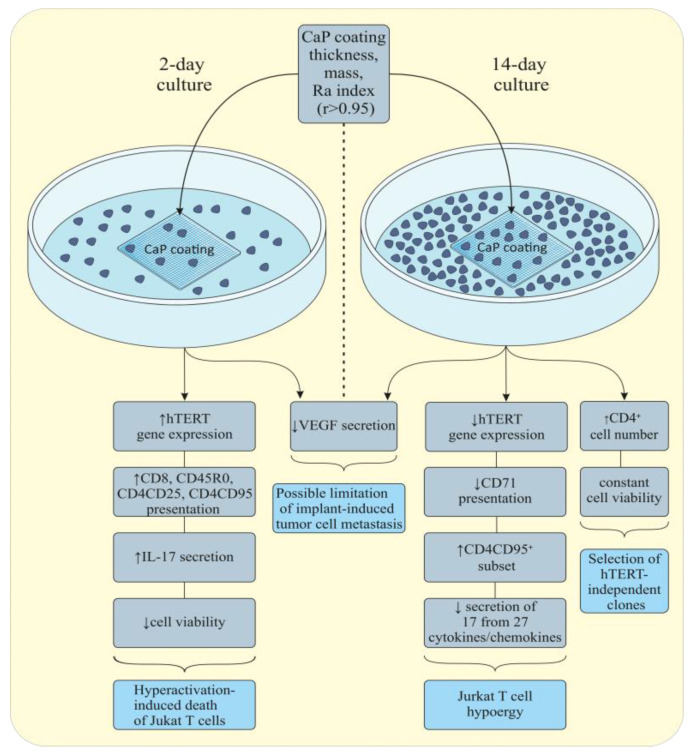
Variable reactions of Jurkat T cells induced by rough microarc CaP-coated titanium substrates in short-term and long-term cultures.

Thus, the variable morphofunctional response of widely used Jurkat T cells cocultured with CaP-coated samples should be considered when testing materials that are meant to have durable contact with the human body, especially in cancer patients. The main results are presented in Figure 7. Therefore, many in vitro estimations of implant biological properties may change in long-term cell cultures.

## 5. Conclusions

This study showed voltage-dependent (150–300 V) growth of structural (*R_a_* index, mass, and thickness) and morphological (spherulites, valleys, and pores) surface and volume elements, low calcium content (Ca/P_aT_ = 0.3–0.6), the appearance of crystalline phases of CaHPO_4_ (monetite) and β-Ca_2_P_2_O_7_ (calcium pyrophosphate), and an increased magnitude of negative electrostatic voltage in the CaP coatings deposited via MAO on Ti substrate. Strong correlations were identified between technological parameters (*R_a_*, thickness, and mass values), as well as between the index *R_a_* = 2–5 µm and the cellular and molecular behavior of Jurkat T cells cultured in vitro.

Because of the close relations of *R_a_* indices to cytological properties, we distinguished the surface roughness of the microarc CaP coating as a trigger of hyperactivation-dependent death in CD4^+^ cells in 2-day cultures, followed by the selection of *hTERT*-independent viable subsets of Jurkat T cells combined with significant secretory (17 of 27 cytokines/chemokines, including VEGF) and proliferation gene (*hTERT*) hypoergy after prolonged 14-day contact with test samples. Negative ζ-potential and Ca^2+^ could mediate the biological effects of CaP roughness on cell reactions. Impaired mitotic/costimulatory/antiapoptotic signaling of CD71, IL-1, IL-4, IL-6, IL-8, and other chemokines induced by CaP coatings might affect the prolonged survival of Jurkat T cells with downregulated *hTERT* expression. At the same time, the molecular mechanisms underlying the 14-day selection of a portion of CD4^+^ Jurkat T cells in the long-term presence of rough and soluble CaP coating can be further explored.

In summary, reduced chemokine and VEGF secretion by malignant cells correlated with the *R_a_* roughness index of microarc CaP coating is an effective competitive advantage that may distinguish this material for endoprosthetics and osteosynthesis in patients suffering from lymphoid leukemia.

## Figures and Tables

**Table 1:** Monoclonal antibody panel used for the estimation of Jurkat T cell viability and antigen profiles.

Studied Cell	Fluorochromes and Labeled Monoclonal Antibodies				
	FITC	VioBlue	PE	APC	PE-Cy7
Jurkat T cells	Annexin V CD45RA CD95	CD45 CD3	CD8 CD45RO	CD25 CD71	CD4

**Table 2:** *hTERT* expression in Jurkat T cells after different culture times in the presence of a CaP coating on a titanium substrate; Me (Q_1_; Q_3_).

Bilateral CaP Coating			*hTERT*, a.u.
*R_a_*, µm	Thickness, µm	Mass, mg	
2-day culture, *n* = 7			
2.88 (2.22; 3.90)	40.5 (24.0; 58.0)	12.0 (5.2; 17.1)	1.66 (1.15; 1.90)
14-day culture, *n* = 6			
2.96 (2.14; 3.40)	47.5 (32.0; 62.5)	12.8 (9.4; 16.9)	−3.26 (−5.57; −2.34) *

Note: Here, and below, *n* = the number of wells (samples) in each group; * = statistical significance (*p* < 0.05) compared with the 2-day culture according to the Mann–Whitney *U* test; a.u. = arbitrary units; (−) = a suppression of relative gene expression compared with control cell culture without samples; gene measurements were performed in triplicate.

**Table 3:** Viability and immunophenotype of CD45^+^CD3^+^ Jurkat T cells after different culture periods in the presence of the CaP-coated Ti samples; Me (Q_1_; Q_3_).

Bilateral CaP Coating			Live or Dead Cells, %			Cells Expressing Specific Membrane Markers, %						
*R_a_*, µm	Thickness, µm	Mass, mg	Viable Cells	Apoptosis	Necrosis	CD4	CD8	CD71	CD45RA	CD45R0	CD4CD25	CD4CD95
(1) Cells on plastic surface (control) after 2-day culture, *n* = 4												
-	-	-	84.95 (82.95; 86.55)	4.05 (2.40; 6.0)	11.05 (11.0; 11.1)	95.10 (95.03; 95.54)	1.44 (0.95; 2.22)	75.42 (71.90; 75.74)	91.97 (91.44; 92.33)	1.81 (1.42; 1.96)	17.30 (15.61; 17.98)	83.01 (82.48; 83.61)
(2) Cells in contact with the CaP-coated Ti samples after 2-day culture, *n* = 7												
2.88 (2.22; 3.90)	40.5 (24.0; 58.0)	12.0 (5.2; 17.1)	76.3 (70.0; 77.2) P_1_ < 0.002	8.0 (7.2; 10.5) P_1_ < 0.04	16.3 (15.6; 18.1) P_1_ < 0.001	93.93 (92.39; 94.85)	3.60 (3.30; 4.96) P_1_ < 0.003	74.54 (72.06; 76.62)	81.70 (80.0; 89.19) P_1_ < 0.02	7.08 (2.93; 10.11) P_1_ < 0.04	20.0 (16.95; 22.52) P_1_ < 0.02	88.18 (84.75; 89.59) P_1_ < 0.003
(3) Cells on plastic surface (control) after 14-day culture, *n* = 3												
-	-	-	90.92 (90.18; 91.98)	2.88 (2.79; 2.99)	6.29 (5.14; 6.83)	62.16 (59.78; 62.8)	2.77 (2.51; 3.77)	98.97 (98.56; 99.24)	99.3 (99.23; 99.34)	37.49 (33.42; 38.81)	27.88 (26.66; 29.04)	39.03 (38.28; 45.24)
(4) Cells in contact with the CaP-coated Ti samples after 14-day culture, *n* = 6												
2.96 (2.14; 3.40)	47.5 (32.0; 62.5)	12.8 (9.4; 16.9)	92.32 (89.6; 92.79)	2.45 (1.81; 2.84)	5.87 (4.76; 7.56)	77.65 (77.15; 81.11) P_3_ < 0.001	0.61 (0.41; 0.81) P_3_ < 0.001	95.92 (95.6; 96.35) P_3_ < 0.001	99.15 (99.12; 99.41)	39.05 (35.91; 41.05)	28.77 (27.93; 35.32)	61.92 (57.27; 62.04) P_3_ < 0.002

Note: P_n_, significant difference (<0.05) compared with the corresponding group number according to the Mann-Whitney *U* test. Duplicate probes for each well were measured.

**Table 4:** Cytokine concentrations (pg/mL) in the supernatants of Jurkat T cells after different culture times in the presence of the CaP-coated Ti samples; Me (Q1; Q3).

Bilateral CaP Coating			Inflammatory Interleukins and Cytokines														
*R_a_*, µm	Thickness, µm	Mass, mg	IL-1β	IL-1Ra	IL-2	IL-4	IL-5	IL-6	IL-7	IL-9	IL-10	IL-12(p70)	IL-13	IL-15	IL-17	TNFα	IFNγ
Nutrient medium without cells, *n* = 3																	
-	-	-	0.02 (0.01; 0.05)	0.05 (0.03; 0.80)	0 (0; 0.53)	0 (0; 0)	0 (0; 0)	0 (0; 0.35)	0 (0; 0)	0 (0; 0)	0.01 (0.01; 0.23)	0 (0; 0)	0 (0; 0)	0 (0; 0)	0 (0; 0)	0 (0; 0)	0 (0; 0)
1) Cells on plastic surface (control) after 2-day culture, *n* = 3																	
0	0	0	0.46 (0.43; 0.63)	7.65 (3.23; 8.65)	1.90 (0.92; 2.11)	0.21 (0; 0.3)	2.87 (1.25; 3.22)	2.39 (2.03; 3.12)	0 (0; 0)	1.72 (0.99; 1.98)	9.90 (9.63; 10.46)	4.89 (3.45; 5.52)	1.48 (1.26; 2.12)	5.49 (4.38; 6.55)	2.21 (1.03; 2.09)	6.09 (3.29; 10.23)	0 (0; 0)
(2) Cells in contact with the CaP-coated Ti samples after 2-day culture, *n* = 7																	
2.88 (2.22; 3.90)	40.5 (24.0; 58.0)	12.0 (5.2; 17.1)	0.43 (0.3; 0.56)	3.36 (1.10; 5.43)	1.90 (1.43; 2.22)	0.24 (0; 0.43)	2.06 (0.99; 3.26)	2.53 (1.90; 2.98)	0 (0; 0)	2.66 (2.29; 3.04)	9.08 (7.87; 9.63)	3.78 (2.75; 5.70)	1.26 (1.04; 1.80)	4.90 (4.44; 6.11)	5.39 (3.72; 6.78) P_1_ < 0.05	4.03 (3.30; 5.40)	0 0; 0)
(3) Cells on plastic surface (control) after 14-day culture, *n* = 5																	
0	0	0	0.37 (0.36; 0.37)	40.08 (36.56; 45.06)	0.92 (0.68; 1.37)	1.0 (0.67; 1.09)	0 (0; 0.54)	487 (479; 491)	1.94 (1.81; 3.03)	3.88 (3.31; 4.32)	19.58 (19.29; 20.39)	88.52 (88.45; 89.48)	2.44 (1.94; 2.57)	0 (0; 0)	1.20 (0.43; 2.52)	9.99 (9.03; 12.81)	51.16 (29.56; 53.73)
(4) Cells in contact with the CaP-coated Ti samples after 14-day culture, *n* = 6																	
3.3 (2.7; 5.0)	54.0 (41.5; 73.0)	14.6 (11.5; 19.0)	0.12 (0.01; 0.19) P_3_ < 0.001	7.82 (2.92; 15.39) P_3_ < 0.001	0.93 (0.69; 1.07)	0.45 (0.30; 0.54) P_3_ < 0.001	0 (0; 0)	0.97 (0.52; 1.22) P_3_ < 0.001	1.76 (1.28; 2.39)	2.87 (2.51; 3.30) P_3_ < 0.006	10.73 (8.62; 13.39) P_3_ < 0.001	46.15 (23.46; 54.13) P3 < 0.001	1.37 (0.80; 1.77) P_3_ < 0.02	0 (0; 0)	0.65 (0; 1.50)	3.78 (2.06; 5.41) P_3_ < 0.001	10.79 (3.43; 20.45) P_3_ < 0.01
**Bilateral CaP Coating**			**Angiogenic Molecules**			**Hematopoietic Growth Factors**			**Chemokines**								
***R_a_*,** **µm**	**Thickness, µm**	**Mass,** **mg**	**bFGF**	**VEGF**	**PDGF-BB**	**G-CSF**	**GM-CSF**		**IL-8** **(CXCL8)**		**Eotaxin** **(CCL11)**	**IP-10** **(CXCL10)**	**MCP-1** **(CCL2)**	**MIP-1α** **(CCL3)**	**MIP-1β** **(CCL4)**	**RANTES** **(CCL5)**	
Nutrient medium without cells, *n* = 3																	
-	-	-	0.91 (0; 1.56)	0 (0; 0)	0 (0; 0)	0 (0; 0)	1.29 (0.09; 1.92)		0 (0; 0)		0 (0; 0)	0 (0; 0)	0 (0; 0)	0 (0; 0)	0 (0; 0)	0 (0; 0)	
(1) Cells on plastic surface (control) after 2-day culture, *n* = 3																	
0	0	0	6.54 (6.24; 10.32)	94.4 (81.6; 108.9)	0 (0; 0)	0 (0; 0)	93.2 (86.6; 95.6)		3.22 (3.20; 6.12)		1.03 (0; 2.09)	5.22 (4.20; 5.22)	6.49 (5.16; 7.13)	0.43 (0.43; 1.07)	2.34 (1.25; 2.50)	3.20 (2.89; 5.34)	
(2) Cells in contact with the CaP-coated Ti samples after 2-day culture, *n* = 7																	
2.88 (2.22; 3.90)	40.5 (24.0; 58.0)	12.0 (5.2; 17.1)	9.96 (8.80; 15.60)	60.1 (48.7; 61.9) P_1_ < 0.001	0 (0; 0)	0 (0; 0)	121 (114; 133) P_1_ < 0.001		10.82 (5.51; 13.08)		0 (0; 0.38)	2.19 (0.98; 3.80)	18.8 (12.4; 20.8) P_1_ < 0.003	4.24 (1.96; 4.62) P_1_ < 0.01	65.8 (30.7; 70.5) P_1_ < 0.003	3.45 (2.34; 8.33)	
(3) Cells on plastic surface (control) after 14-day culture, *n* = 5																	
0	0	0	14.48 (14.47; 17.95)	1735 (1707; 1768)	2.16 (2.14; 3.83)	43.77 (39.28; 45.67)	58.94 (55.33; 59.20)		47.69 (47.57; 47.70)		10.49 (9.97; 10.79)	21.27 (19.49; 22.53)	19.55 (18.57; 20.02)	0.29 (0.25; 0.39)	4.58 (4.58; 4.63)	1.32 (1.26; 1.52)	
(4) Cells in contact with the CaP-coated Ti samples after 14-day culture, *n* = 6																	
3.3 (2.7; 5.0)	54.0 (41.5; 73.0)	14.6 (11.5; 19.0)	11.32 (8.61; 12.48)	299 (224; 392) P_3_ < 0.001	1.81 (0.87; 2.41)	2.63 (1.28; 3.76) P_3_ < 0.001	48.79 (40.19; 55.40)		3.17 (2.66; 3.65)P_3_ < 0.001		3.21 (1.93; 4.53) P_3_ < 0.001	13.72 (11.79; 15.95) P_3_ < 0.002	6.28 (5.54; 7.10) P_3_ < 0.001	0.28 (0.26; 0.30)	5.26 (4.94; 5.54)	0.71 (0; 0.98) P_3_ < 0.003	

Note: P_n_, significant difference (<0.05) compared with the corresponding group number according to the Mann–Whitney U test. Duplicate probes for each well were measured.
